# A spectrum of self-processing modes: Indian philosophical insights and contemporary science on wellbeing

**DOI:** 10.3389/fpsyg.2026.1884131

**Published:** 2026-07-07

**Authors:** Sonu Sharma, Pradeep Kumar, Vishva Chaudhary, Anuranjan Kumar, Anupam Yadav, Ravinder Kumar, Annu Yadav, Anshul Girdhar, Nidhi Kalonia, Pavitra Singh

**Affiliations:** 1 Department of Psychology, Central University of Haryana, Mahendergarh, Haryana, India; 2 Department of Psychology, Central University of Karnataka, Kalaburagi, Karnataka, India; 3 Department of Psychology, Pavitra Degree College, Gorakhpur, Uttar Pradesh, India; 4 Department of Psychology, Amity University, Gurugram, Haryana, India

**Keywords:** Advaita Vedanta, anatta, choiceless-awareness, consciousness, default mode network, non-dual awareness, wellbeing

## Abstract

While contemporary psychology focuses on developing a cohesive self for wellbeing, Indian philosophical traditions such as Advaita Vedanta, Buddhism and the teachings of J. Krishnamurti provide a critique of the notion of a fixed self-identity. This conceptual paper compares them at a phenomenological and functional level and relates these comparisons to contemplative neuroscience, particularly the default mode network, self-referential processing, and meditation-related changes in self-experience. Furthermore, It proposes a spectrum model: self-referential processing as an ordinary mode, meta-awareness as a trainable capacity, and non-self experience as a transformed outcome. The framework suggests that wellbeing may involve flexible regulation of self-identification rather than simple self-enhancement. Finally, implications for research, clinical practice, and cultural interpretation are discussed.

## 1 Introduction

Human beings across cultures have long sought ways to alleviate suffering and promote wellbeing. Contemporary Western psychology has primarily approached this goal by strengthening a coherent and positive sense of self. While these models have proven fruitful, they may also limit the range of psychological experiences considered meaningful and adaptive (Ge and Yang, 2023; Wong et al., 2021). Indian philosophical traditions such as Buddhism, Advaita Vedanta, and the teachings of J. Krishnamurti challenge the notion of egoic-self. Rather than strengthening the ego, these traditions view the sense of a separate self as the root of psychological suffering and propose that wellbeing arises from insight into the constructed and impermanent nature of the self (Collins, 1982; Harvey, 2013; Shiah, 2016). These traditions offer an alternative framework for understanding consciousness and the emergence of psychological suffering. This perspective shifts attention toward examination of the constructed nature of egoic identity.

In this paper, the term non-self is used as a phenomenological and process-level descriptor, referring to experiential configurations characterized by reduced identification with narrative and ego-centered processes. It does not assert a metaphysical position about whether the self exists or not. The central question is therefore not whether the self exists in some absolute sense, a question on which Buddhism, Advaita Vedanta, and Krishnamurti differ profoundly, but rather how different modes of self-experience relate to psychological suffering, regulation, and wellbeing (Shiah, 2016; Wong et al., 2021). The three traditions are compared here not at the metaphysical level but at the level of phenomenological convergence: their shared functional diagnosis that attachment to a reified ego-center produces suffering and that its attenuation is associated with transformed wellbeing (Thompson, 2015; Varela et al., 2016; Loy, 1982).

The paper is organized as follows: Section 2 reviews contemporary psychological and neuro-cognitive understandings of the self; Section 3 examines the philosophical foundations across the three traditions and their points of phenomenological convergence; Section 4 proposes the spectrum model and its neural correlates; and Section 5 discusses theoretical, methodological, and clinical implications.

## 2 Defining the conceptual construct of self

Contemporary psychology and cognitive neuroscience identify multiple layers and modes of self-representation. One of the most prominent distinctions is the one made between the minimal self and the narrative self. The minimal self refers to a pre-reflective awareness of oneself as the immediate subject of experience, an implicit first-person perspective grounded in embodied and sensorimotor mechanisms (Gallagher, 2000, 2013). The narrative self, by contrast, involves extended autobiographical and prospective representations that support a coherent sense of personal identity over time (Horváth, 2024; pp, 37–86). Narrative forms of self-representation are closely associated with self-referential processing and with activity in large-scale brain networks such as the default mode network. Self-referential processing is the neural and cognitive activation associated with thinking about oneself—emerges from distributed systems involving memory, attention, emotion regulation, and interoception (Buckner et al., 2008; Davey et al., 2016; Spreng and Grady, 2010; Northoff et al., 2006).

Studies on self-transcendence and decentering show that there can be changes in the extent to which thoughts, feelings and perceptions are identified as “self” (Shiah, 2016; Hanley et al., 2020; Wong et al., 2021). Likewise, models in contemplative science describe states where the self is experienced with altered metacognitive monitoring and self-referential processing, enabling a less identified experience (Dorjee, 2016). Furthermore, neuroscience also demonstrates that self-referential processing can be altered, with advanced meditation practices being associated with changes in the DMN, reduced identification with narrative self-content, and in advanced practitioners states sometimes described as non-dual awareness (Brewer et al., 2011; Garrison et al., 2015; Josipovic, 2014; Ward, 2026).

This process-oriented view of the self is, in some ways, consistent with a number of philosophical traditions that deny the existence of, enduring self. For example, Buddhism views the experience of a seemingly permanent, separate self as the result of misidentification with impermanent aggregates (skandhas). A process that Krishnamurti similarly describes as psychological conditioning around an illusory observer-center. Advaita Vedanta approaches the problem a little differently: it does not deny Atman, but rather locates the source of suffering in the misidentification of individual consciousness (jiva) with limited egoic patterns, rather than with its true nature as Brahman (Thibaut, 1904; Rajagopal, 2024).

## 3 Indian philosophical foundations

Indian philosophical traditions offer a markedly different perspective on wellbeing from mainstream Western psychology (Cornelissen et al., 2011). In these traditions, wellbeing is associated with a transformed mode of experiencing the self where the rigid identification with egoic identity is diminished (Yaden et al., 2017; Shiah, 2016). According to these traditions, transcending self-identification may give rise to states characterized by equanimity, clarity, and compassion (Shiah, 2016; Yaden et al., 2017).

Liberation is not understood as acquisition of something new. It is described as recognizing what has always been present. It involves seeing through the misidentification between one's true nature and the impermanent layers of personality and experience (Radhakrishnan, 1953; Rajagopal, 2024; Thibaut, 1904). In this view, awakening is conceptualized not as an achievement, but as a transformation in understanding, where the illusion of separation fades.

Contemporary interpretations sometimes misunderstand this idea of liberation or non-self, assuming it implies nihilism or a lack of experience. In the Indian traditions, however, it refers to a more expansive and liberated form of awareness, not its absence.

### 3.1 Buddhist understanding of self

Buddhism approaches human experience quite differently from how Western psychology tends to frame it. Instead of assuming a fixed self that needs to be understood or strengthened, Buddhist philosophy breaks down what we typically call “self” into five components known as Skandhas or aggregates. These five categories—Form (rupa), Sensation or Feeling-tone (vedana), Perception and Conceptualization (sañña), mental formations including volitions and emotions (samskara), and consciousness itself (vijnana)—constitute everything we experience as ourselves (Harvey, 2013; Siderits, 2003).

A central feature of this perspective is that none of these five components remains constant. The body continuously changes. Sensations arise and vanish. Perceptions shift as we interpret different experiences. Mental formations—our thoughts, feelings, habitual patterns—constantly transform. And consciousness itself, far from being a unified observer, is itself a dependent phenomenon. From this perspective, there's no permanent, unchanging “I” running through all these experiences. There are momentary perceptions and reactions, but no solid entity that observes them from outside (Collins, 1982; Harvey, 2013).

#### 3.1.1 Suffering as rooted in attachment

Within Buddhist psychology, attachment (tanha) emerges directly from false identification with skandhas. When you believe yourself to be a limited, vulnerable individual separate from the world, you naturally develop an urgent need to protect and validate this imagined entity. You crave experiences that seem to strengthen it—approval, possessions, status, pleasures. Simultaneously, you develop aversion to experiences that threaten it—criticism, loss, low status, pain. These cravings and aversion aren't merely psychological reactions; they're the natural consequences of identifying with something temporary and inherently insecure (Harvey, 2013).

#### 3.1.2 Nirvana and cessation of suffering

Buddhist philosophy proposes that when direct experiential insight arises into the impermanent and conditioned nature of the five aggregates (skandhas), the processes of misidentification that sustain psychological suffering begin to weaken (Harvey, 2013; Shiah, 2016). In this condition, the person is still, the Body is still, Sensations are still and Consciousness is still with all its natural changes and functioning. But now there is no longer a separate self-identity, separate “I.” This is not a change or an upgrade of the current self-structure but a different type of realization into being (Harvey, 2013; Shiah, 2016).

### 3.2 Advaita Vedanta

The philosophy of Advaita Vedanta was popularized by the 8th-century Indian philosopher Adi Shankaracharya. This view makes a far more radical claim that there is only one ultimate reality (Thibaut, 1904). This reality is termed as Brahman. It is described as pure, limitless consciousness. Everything else the appearance of many selves, the sense of being a separate individual, and the entire world of multiplicity arises only from ignorance (avidya) and the play of illusion known as maya (Thibaut, 1904)

In this framework, what we normally call the individual self as the thinker, the doer, the person who feels and experiences has no independent existence. The familiar sense of “I,” experienced as a separate person with distinct boundaries, appears real only because consciousness misidentifies itself with the body and mind (Rajagopal, 2024; Thibaut, 1904).

#### 3.2.1 Maya and Avidya as the source of separation

Maya is understood as distorted reality and not unreal existence. It means that the world is relatively real. One of the ways Indian scholars put it is by recognizing two types of reality: first, the paramarthika satya (transcendental reality) where only Brahman exists and second, the vyavaharika satya (practical reality) where the world and individuals are. When consciousness identifies itself with the apparent individual self, there are certain psychological implications. This apparent distinction creates what Advaita refers to as the basic dualistic view: the feeling of being a separate entity among a multiplicity of other separate entities (Thibaut, 1904).

#### 3.2.2 Self-recognition as liberation

Self recognition in Advaita Vedanta conceptualized as opening of the eyes to the truth that has always been there. It is realizing the fact that one's essential nature (Atman) is identical with Brahman, the ultimate, infinite, unchanging reality (Thibaut, 1904). According to (Radhakrishnan 1953) “Tat Tvam Asi” points to this idea only that means that the individual imagines oneself to be is in fact identical with ultimate reality.

This realization, often described as enlightenment is the realization that dissolves the illusion of separation. It is when consciousness recognizes its own non-dual nature, when it perceives directly, not merely intellectually but experientially then, there is only one consciousness appearing as the multiplicity of individual consciousnesses. The fear born from perceiving oneself as separate ends (Thibaut, 1904).

### 3.3 Krishnamurti's radical approach to psychological transformation

Krishnamurti's view of psychological conditioning goes deeper than the behaviorist idea of learning through stimuli and responses. He noted that methods built on reward and punishment work only at a surface level. They may change behavior, but they do not address the roots of psychological suffering. In his understanding, conditioning includes everything that shapes a person's inner world: beliefs, memories, habits, social roles, and the entire sense of who one thinks they are (Krishnamurti, 1969). These patterns are shaped continuously by family, culture, and society, and together they form a structure that creates and sustains inner conflict (J. Krishnamurti, 1969).

Krishnamurti emphasized that trying to adjust or improve this structure of the self only rearranges the same conditioning (Krishnamurti, 1969; Rao, 1995). It does not address the quality of mind that produced disorder in the first place. Krishnamurti argued that psychological suffering does not arise merely from problems within the self, but from identification with the self as a psychological center. Any positive ideas or self-improvements do not dissolve the core of suffering, which he described as the split between the observer (the self) and the observed (experience) (Krishnamurti, 1969).

#### 3.3.1 Choice-less awareness

The central teaching of Krishnamurti's work is what he called “choice-less awareness”—a state of simple, steady observation. The state- where the mind isn't filtered through thought, preference, or the constant urge to control experience (Krishnamurti, 2000). Here, we have no central agent, we call “the Self.” In such a state, there is no distinction between the two, for example, between an observer and thoughts or feelings. An observation is immediate and encompasses everything surrounding a person because there is no mental center which evaluates and influences further events (Krishnamurti, 1969). Krishnamurti understands freedom as not the ability to satisfy one's individual interests but liberation from oneself who acts according to the principle of the established center. Such a center, or “I,” is the one that makes comparisons, creates fear, generates desires, and aims at security. In turn, it leads to the separation of the observer from the object of observation, thus sustaining the vicious circle of conflicts and sufferings (Krishnamurti, 1969). It will stop when one fully concentrates on the present moment, which, however, can happen involuntarily.

#### 3.3.2 Transformation vs. adjustment

The progression of the self to no-self can be understood in the context of a type of psychological transformation. It is not a quantitative improvement of the self into a better self, but rather a qualitative transformation. The idea proposed by Krishnamurti was that as long as there was a separation between the “observer” (the part of oneself that observes and tries to change) and the “observed” (thoughts, emotions, or behaviors), there would be continuous conflict and suffering (Krishnamurti, 1970).

It is significant to note that for Krishnamurti, direct realization of the notion “the observer is the observed” would imply that the thinker and thought were identical. This way, a basic foundation of psychological struggle disappears (Krishnamurti, 1954). As a result, actions directed toward self-improvement or emotional regulation do not make any sense. There is a dissolution of the basic division between the self and its experience, which leads to a new kind of coherent and united perception (Krishnamurti, 1954). What is especially important about Krishnamurti's approach was that the insight of no “me” who accepts, controls, or changes experience meant an elimination of all psychological conflicts.

### 3.4 Convergence and divergence among these traditions

Though these traditions seem to be the similar, Buddhism, Advaita Vedanta and Krishnamurti have a significant difference at the metaphysical level. Buddhism rejects the concept of a permanent self and sees freedom as the direct realization of the absence of a permanent self or the ego-entity (Collins, 1982; Harvey, 2013).

In contrast, Advaita Vedanta does not reject the concept of self in its entirety, but rather states that the real self is the pure and eternal consciousness, which is Atman, while the misconception of self is with regard to the limited self, namely the egoic personality, and not the real self, namely Atman, which is infinite, in its true state as Brahman (Thibaut, 1904; Rajagopal, 2024).

Krishnamurti reaches a similar conclusion, a structurally parallel one, by means of a direct psychological questioning, not by means of a doctrine. Krishnamurti, who is neither dogmatic in his adherence to a tradition, nor sees any barriers to the destruction of the individual, defines liberation as the disappearance of the observer–observed dichotomy and the cessation of the psychological ‘self', as a center of reference established by thoughts (Krishnamurti, 1969).

The present paper does not argue the doctrinal equivalent for these three traditions nor does it try to make a metaphysical synthesis. Instead, the comparison is restricted to what (Thompson 2015) and (Varela et al. 2016) refer to as phenomenological convergence: the proximal claim, whether or not it has a metaphysical basis, that attachment to the egoic-identity, is a proximate cause of psychological distress and that its reduction through practice is correlated with a reduction of distress and an increase in psychological wellbeing. This is often referred to as neurophenomenology, since it allows for the comparison of traditions across disciplines without the need to reconcile their metaphysical implications (Varela et al., 2016). The three traditions are brought together at this level, the phenomenology of the egoic misidentification as suffering and the weakening of it as liberation.

## 4 A spectrum of self-processing modes: neural and phenomenological correlates

A growing body of contemplative and neuroscientific research now recognizes a fluid picture of self. It suggests that our experience of self is not fixed but can be understood across a spectrum of modes, capacities, and outcomes that vary in degree of identification with narrative self-processes (Shiah, 2016; Dorjee, 2016). This section brings together insights from contemplative neuroscience, particularly research on the default mode network (DMN), with the profound observations found in Indian philosophical traditions to propose a Spectrum of Self-Processing Modes—a framework that offers a fresh way of understanding how different modes of self-experience relate to psychological suffering and wellbeing.

### 4.1 Self as a product of evolution

The human sense of self is an evolutionary adaptation that emerged to enhance survival through better prediction, social coordination, and threat management (Skowronski and Sedikides, 2019; Grinde, 2024). Research shows that the self with multiple cognitive systems evolved over hundreds of thousands of years (Skowronski and Sedikides, 2019). This executive function capacity allowed early humans to recall past threats, anticipate future dangers, and coordinate behavioral responses, a significant adaptive advantage (Barkley, 2001; Skowronski and Sedikides, 2019).

The adaptive capacity that evolved for survival has now, under conditions of chronic activation, become a source of psychological difficulty. As humans are now able to predict the future, they are now able to predict future threats, which creates tension in real time. it also provides them an insight that they are mortal. This insight created a sense of anxiety in the human beings. This anxiety, by researchers termed as existential anxiety (Ge and Yang, 2023; Yaden et al., 2017).

### 4.2 Relationship to contemporary research

Emerging research in contemplative science on mindfulness and meditation suggest that self-related processing is not fixed but can be modulated through attentional and metacognitive training (Dorjee, 2016). For example, increased connectivity and altered dynamics across large-scale brain networks—including the default mode, salience, and executive networks—have been associated with meditation practice, indicating changes in how internally generated experience is organized (Bremer et al., 2022; Brewer et al., 2011). The Default Mode Network and Modes of Self-Processing

The default mode network (DMN) refers to a set of brain regions commonly associated with internally directed cognition, including autobiographical reflection and self-referential thought. Brain imaging studies using fMRI and MEG show that when we are simply sitting quietly, these DMN regions are constantly in conversation with each other in slow, rhythmic patterns of activity (Buckner et al., 2008; Northoff, 2025). This network shows increased activity during autobiographical remembering, and self-referential narrative processing, processes often associated with construction of personal identity (Schacter et al., 2012; Spreng and Grady, 2010). In this way, these processes are thought to contribute to what researchers describe as the narrative self, an identity composed of memories and projections of the future (Northoff et al., 2006; Schacter et al., 2012). This internal narration shapes our sense of who we are (Spreng and Grady, 2010). An identity, woven together from past memories and imagined futures (Northoff et al., 2006; Schacter et al., 2012).

Some Neuroscientific models use concepts such as “self-binding” to describe how distributed processes may contribute to the experience of being a unified person. Neuroimaging research consistently shows that self-relevant information is processed with greater efficiency and neural coordination than non-self-relevant information (Spreng and Grady, 2010). Neuroscientific frameworks suggest that the sense of a coherent ‘I' may emerge from distributed cognitive processes (Northoff et al., 2006; Shiah, 2016). When people think about themselves, communication intensifies between the ventromedial prefrontal cortex and other regions handling emotion, attention, and bodily awareness (Northoff et al., 2006). This coordinated pattern of activity has been associated with the subjective experience of continuity across time. Some neuroscientific models propose that the experience of self-unity may depend on coordinated activity across distributed neural systems and not from any permanent, unchanging self-sitting at the center of it all. These findings have been interpreted by some scholars as broadly compatible with Buddhist accounts of non-self (Collins, 1982; Harvey, 2013).

### 4.3 Meditation-related DMN deactivation and the experience of non-self

Advanced forms of meditation, often lead to a noticeable quieting of the default mode network. Studies show that experienced meditators tend to display lower DMN activity both during practice and even when they are simply resting (Bremer et al., 2022; Brewer et al., 2011). These shifts are closely linked with reduced mind-wandering and less habitual rumination (Bremer et al., 2022; Brewer et al., 2011). Mindfulness is mainly used to anchor attention and insight-oriented practices may influence cognitive and neural processes associated with self-referential experience (Brewer et al., 2011). Vipassana in practice requires the subject to closely and uncritically monitor their present experience and there have been numerous reports of an improved state of wellbeing with reduction in activity in self-focused thoughts (Brewer et al., 2011; Giridharan et al., 2025). Intense ten-day practice at a meditation retreat seemed to lead to improved results and more durable results with reduction of stress and anxiety over shorter programs (Giridharan et al., 2025).

### 4.4 Mental health benefits: less rumination, greater wellbeing

Psychologically, it is beneficial for subjects not to be engaged in self-focused thought. (Hamilton et al. 2011) demonstrated that disturbances in the default mode network activity are seen in a number of psychiatric disorders, such as major depression, anxiety and post-traumatic stress disorder (PTSD). These disorders tend to have increased connectivity within the DMN, and increased activity in the posterior cingulate cortex. This is consistent with the rumination process, where the mind becomes stuck in repetitive and self-referential thinking that reinforces negative beliefs and negative feelings (Hamilton et al., 2011).

Rumination has been associated with persistent self-referential processing and altered default mode network dynamics. Mind wanders to the past or to perceived future threats and the self is seen as vulnerable and inadequate. The activation of the DMN becomes greater and greater with these states and therefore so do the negative narratives.

In all, these insights suggest that the self can be conceived in terms of a spectrum of self-processing mode, from highly self-referential and narrative forms to states with less identification with self-related phenomena. This conceptual space offers a bridge between psychological theories of self-regulation and philosophical theories of non-self. It offers the possibility that what is claimed in contemplative practice to be non-self may correspond, at least in part, to certain patterns of cognitive and experiential processes rather than the simple absence of self (Ward, 2026). Hence, this spectrum can be conceptualized in 3 components:

### 4.5 The following is the order of self-processing modes

**(A). Self-referential processing:** It refers to the ordinary mode of self-related cognition, characterized by dominant narrative identity, autobiographical processing, and ego-centered evaluation. This mode is understood as functionally adaptive and is not inherently pathological. It becomes problematic when chronically activated in the form of rigid, intrusive rumination (Nolen-Hoeksema et al., 2008).

**(B). Meta-Awareness: Second** order skill that may be cultivated by practices like mindfulness, Vipassanā, and contemplative inquiry (Lutz et al., 2015). As (Dorjee 2016) and (Vago and Silbersweig 2012) have suggested this capacity is a link between ordinary self-referential processing and transformed self-transcendent experience in that it allows for a less fused, less automatic connection to the self-referential cognitive content.

**(C). Non-self:** Self transcendent experience is an experiential outcome that is marked by a decrease in the identification of processes of the narrative self, a decrease in the subject–object structuring, and the phenomenological states which are variously described across the traditions as anattā, non-dual awareness, or choiceless awareness.

This is described here as an experience qualitatively different than any other and is the emergence of a capacity for meta-awareness, when it is sufficiently developed and maintained.

The model thus separates out three modes of experience, an ordinary experiential, a mediating capacity, and a transformed experiential, that are related to each other in a developmental way (Table 1).

**Table 1:** Spectrum of self-processing modes.

Mode	Phenomenology	Cognition	Neuroscience	Clinical relevance
**Self-referential** (ordinary mode) ↕	Strong ‘I' involvement; narrative self salient. Sense of continuous, bounded personal identity.	Autobiographical recall, evaluation, comparison, future-past simulation. Adaptive for planning & social coordination.	DMN-dominant; high vmPFC–PCC connectivity. Elevated self-referential processing (Northoff et al., 2006).	Adaptive baseline. Unnecessary activity of this mode is Vulnerable to chronic rumination & anxiety (Nolen-Hoeksema et al., 2008).
**Meta-awareness** (trainable capacity) ↕	Observing thoughts & emotions without fusion. Reduced automatic reactivity to self-related content.	Decentering; metacognitive monitoring. Second-order awareness of ongoing cognition. Mediates between ordinary selfing & transcendent states.	Reduced dominance of self-referential DMN activity; increased salience & executive network engagement (Bremer et al., 2022).	Core mechanism in MBSR, MBCT, ACT. Measured by EQ (Fresco et al., 2007) & TMS (Lau et al., 2006).
**Non-self** (transformed outcome)	Weak or absent central self-feeling. Non-dual or unified awareness; Minimal subject–object distinction.	Markedly reduced identification with narrative & evaluative processes. Corresponds to anatta, non-duality, or choiceless awareness.	Significant DMN deactivation; reduced narrative/self-bound processing in advanced meditators (Brewer et al., 2011; Garrison et al., 2015).	Associated with reduced suffering & enhanced wellbeing (Yaden et al., 2017). Measured via NADA (Hanley et al., 2018) & STM-Brief (Wong et al., 2021).

The three modes do not share a single categorical axis. Self-Referential designates the ordinary phenomenological mode of self-related cognition. Meta-Awareness designates a trainable metacognitive capacity (skill) that mediates between ordinary self-referential processing and self-transcendent experience. Non-Self designates a transformed experiential outcome that emerges through sustained meta-awareness practice. Arrows indicate developmental direction: cultivated Meta-Awareness facilitates movement toward the Self-Transcendent mode, but the relationship is neither linear nor inevitable. DMN = default mode network; vmPFC = ventromedial prefrontal cortex; PCC = posterior cingulate cortex; EQ = Experiences Questionnaire; TMS = Toronto Mindfulness Scale; NADA = Nondual Awareness Dimensional Assessment; STM-Brief = Self-Transcendence Measure-Brief.

## 5 Discussion

### 5.1 Rethinking psychological research

This framework does not assume that the self is a stable, unitary entity, but rather allows researchers to explore self-experience as a multidimensional process from ordinary self-referential experiences to trained meta-awareness, to transformed self-transcendent experiences and effects. It also suggest scales and assessment instruments for each dimension of self-experience (Dorjee, 2016).

### 5.2 A bridge between traditional wisdom and scientific research

For a very long time, Western science and contemplative traditions have often developed in parallel or even in tension. Some scientists have often regarded contemplative practices as methodologically intractable for empirical study (Davidson and Dahl, 2018), while many contemplative communities viewed science as unable to grasp the deeper purpose of inner transformation. The present framework suggests that self-experience may be more fruitfully understood as dynamic and multidimensional rather than stable and unitary. This paper contributes in this direction by bringing Indian philosophical frameworks into dialogue with empirical and scientific findings through the methodological principle of phenomenological convergence (Thompson, 2015; Varela et al., 2016).

### 5.3 Therapeutic implications

Therapeutic interventions may include these concepts to examine the deeper structures of self-referential consciousness that contemplative and Indian philosophical traditions interpret as contributing to psychological suffering (Harvey, 2013; Rajagopal, 2024; Shiah, 2016). Consider the case of an individual who possesses material comfort and social stability, yet continues to experience a persistent sense of emptiness or psychological incompleteness. Such experiences may require forms of inquiry that extend beyond conventional symptom-management approaches (Wong et al., 2021; Yaden et al., 2017). From this perspective, psychological transformation is approached not merely as treatment or adjustment, but as insight into the nature of self-identification and consciousness (Krishnamurti, 1973; Shiah, 2016).

### 5.4 Methodological challenges

Several validated instruments have been developed to operationalize the constructs in the proposed spectrum. Self-referential processing can be assessed using the Ruminative Responses Scale (Nolen-Hoeksema and Morrow, 1991) and the Self-Reflection and Insight Scale (Grant et al., 2002). Meta-Awareness and decentering can be measured using the Experiences Questionnaire (Fresco et al., 2007) and the Toronto Mindfulness Scale (Lau et al., 2006). Self-transcendent states can be assessed using the Non-dual Awareness Dimensional Assessment (Hanley et al., 2018) and the Self-Transcendence Measure-Brief (Wong et al., 2021). These instruments provide empirically tractable entry points into the spectrum model and suggest a research programme that can test the developmental relationships among the three elements. Still researchers can face a genuine problem to measure these kinds of subjective experiences. In advanced meditation, that includes partial loss of self-referential thinking- makes ordinary self-reporting scales difficult to use. Physiological and neuroimaging tools such as EEG, fMRI, heart-rate variability or breathing patterns can show what happens in the body and brain during deep meditation (Brewer et al., 2011; Davidson and Dahl, 2018). Still, these techniques cannot tell us what the state actually felt like. Because these techniques can only report about the consequences of a certain experience not about the original experience itself. In some advanced practices, practitioners often have very little to report afterward because there was no clear sense of a witnessing self during the event (Davidson and Dahl, 2018). Thus, researchers can observe neural correlates of meditative states, but the subjective dimension remains largely inaccessible to standard measurement.

### 5.5 Differentiating between authentic experience from psychological disturbance

Another significant challenge is to learn how to distinguish between positive experiences of self-transcendence and instances where the self breaks down in a pathological way (Britton et al., 2021; Simeon and Abugel, 2006). At surface level they might appear similar. As they both involve dissolving sense of self. But the related experience and long-term consequences are very different. Authentic non-self experiences are generally marked by clarity, peace, openness, and a sense of connectedness, and are associated with positive long-term effects such as reduced suffering, increased equanimity, and improved wellbeing (Yaden et al., 2017; Wong et al., 2021). Practices of advanced meditation in some people can worsen anxiety, depersonalization or destabilization, particularly when they are undertaken without supervision and without preparation (Britton et al., 2021; Simeon and Abugel, 2006).

## 6 Conclusion

Psychological wellbeing may involve more than developing a stable and coherent sense of self. A functional sense of self remains important for everyday life; however, human experience may not be limited to fixed patterns of self-identification. In certain contemplative contexts, reduced attachment to narrative and evaluative forms of self-experience may be associated with lower psychological suffering and greater inner stability. Rather than treating the self as a rigid or permanent entity, both Indian philosophical traditions and emerging contemplative research point toward a more dynamic understanding of consciousness and identity.

The purpose of this paper is not to claim that neuroscience validates metaphysical doctrines, but to explore possible areas of dialogue between contemplative traditions and contemporary psychological science. In doing so, it highlights the possibility that wellbeing may also involve shifts in how experience is related to, interpreted, and identified with.

By conceptualizing selfhood as a dynamic and multidimensional process, and by differentiating ordinary self-referential processing from the trainable capacity of meta-awareness and the transformative potential of self-transcendent experience, psychology may develop a more nuanced, scientifically rigorous, and culturally inclusive understanding of human flourishing, mental health, and wellbeing.

## References

[B1] Barkley R. A. (2001). The executive functions and self-regulation: an evolutionary neuropsychological perspective. Neuropsychol. Rev. 11, 1–29. doi: 10.1023/A:1009085417776 11392560

[B2] Bremer B. Wu Q. Mora Álvarez M. G. Hölzel B. K. Wilhelm M. Hell E. . (2022). Mindfulness meditation increases default mode, salience, and central executive network connectivity. Sci. Rep. 12:13219. doi: 10.1038/s41598-022-17325-6 35918449 PMC9346127

[B3] Brewer J. A. Worhunsky P. D. Gray J. R. Tang Y.-Y. Weber J. Kober H. (2011). Meditation experience is associated with differences in default mode network activity and connectivity. Proc. Nat. Acad. Sci. 108, 20254–20259. doi: 10.1073/pnas.1112029108 22114193 PMC3250176

[B4] Britton W. B. Lindahl J. R. Cooper D. J. Canby N. K. Palitsky R. (2021). Defining and measuring meditation-related adverse effects in mindfulness-based programs. Clin. Psychol. Sci. 9, 1185–1204. doi: 10.1177/2167702621996340 35174010 PMC8845498

[B5] Buckner R. L. Andrews-Hanna J. R. Schacter D. L. (2008). The brain's default network: anatomy, function, and relevance to disease. Ann. N. Y. Acad. Sci. 1124, 1–38. doi: 10.1196/annals.1440.011 18400922

[B6] Collins S. (1982). Selfless persons: Imagery and Thought in Theravada Buddhism. Cambridge: Cambridge University Press. doi: 10.1017/CBO9780511621499

[B7] Cornelissen R. M. M. Misra G. Varma S. (Eds.). (2011). Foundations of Indian Psychology: Vol. 1. Theories and Concepts. New Delhi: Pearson India.

[B8] Davey C. G. Pujol J. Harrison B. J. (2016). Mapping the self in the brain's default mode network. NeuroImage 132, 390–397. doi: 10.1016/j.neuroimage.2016.02.022 26892855

[B9] Davidson R. J. Dahl C. J. (2018). Outstanding challenges in scientific research on mindfulness and meditation. Perspect. Psychol. Sci. 13, 62–65. doi: 10.1177/1745691617718358 29016238

[B10] Dorjee D. (2016). Defining contemplative science: the metacognitive self-regulatory capacity of the mind, context of meditation practice and modes of existential awareness. Front. Psychol. 7:1788. doi: 10.3389/fpsyg.2016.01788 27909417 PMC5112249

[B11] Fresco D. M. Moore M. T. van Dulmen M. H. M. Segal Z. V. Ma S. H. Teasdale J. D. . (2007). Initial psychometric properties of the experiences questionnaire: validation of a self-report measure of decentering. Behav. Ther. 38, 234–246. doi: 10.1016/j.beth.2006.08.003 17697849

[B12] Gallagher S. (2000). Philosophical conceptions of the self: implications for cognitive science. Trends Cogn. Sci. 4, 14–21. doi: 10.1016/S1364-6613(99)01417-5 10637618

[B13] Gallagher S. (2013). A pattern theory of self. Front. Hum. Neurosci. 7:443. doi: 10.3389/fnhum.2013.00443 23914173 PMC3730118

[B14] Garrison K. A. Zeffiro T. A. Scheinost D. Constable R. T. Brewer J. A. (2015). Meditation leads to reduced default mode network activity beyond an active task. Cogn. Affect. Behav. Neurosci. 15, 712–720. doi: 10.3758/s13415-015-0358-3 25904238 PMC4529365

[B15] Ge B. H. Yang F. (2023). Transcending the self to transcend suffering. Front. Psychol. 14:1113965. doi: 10.3389/fpsyg.2023.1113965 37484086 PMC10361767

[B16] Giridharan S. Soumian S. Kumar N. V. Godbole M. (2025). The impact of Vipassana meditation on health and wellbeing: a systematic review of current evidence. Cureus 17:e93355. doi: 10.7759/cureus.93355 41158896 PMC12558566

[B17] Grant A. M. Franklin J. Langford P. (2002). The self-reflection and insight scale: a new measure of private self-consciousness. Soc. Behav. Personal. 30, 821–836. doi: 10.2224/sbp.2002.30.8.821

[B18] Grinde B. (2024). Consciousness makes sense in the light of evolution. Neurosci. Biobehav. Rev. 164:105824. doi: 10.1016/j.neubiorev.2024.105824 39047928

[B19] Hamilton J. P. Furman D. J. Chang C. Thomason M. E. Dennis E. Gotlib I. H. (2011). Default-mode and task-positive network activity in major depressive disorder: implications for adaptive and maladaptive rumination. Biol. Psychiatry 70, 327–333. doi: 10.1016/j.biopsych.2011.02.003 21459364 PMC3144981

[B20] Hanley A. W. Dambrun M. Garland E. L. (2020). Effects of mindfulness meditation on self-transcendent states: perceived body boundaries and spatial frames of reference. Mindfulness 11, 1194–1203. doi: 10.1007/s12671-020-01330-9 33747250 PMC7968136

[B21] Hanley A. W. Nakamura Y. Garland E. L. (2018). The non-dual awareness dimensional assessment (NADA): new tools to assess non-dual traits and states of consciousness occurring within and beyond the context of meditation. Psychol. Assess. 30, 1625–1639. doi: 10.1037/pas0000615 30058824 PMC6265073

[B22] Harvey P. (2013). An Introduction to Buddhism: Teachings, History and Practices 2nd Edn. Cambridge: Cambridge University Press. doi: 10.1017/CBO9781139050531

[B23] Horváth L. (2024). “The narrative self and the minimal self,” in The Affective Core Self, ed.M. D. Fuchs (Cham: Springer), 37–86.

[B24] Josipovic Z. (2014). Neural correlates of non-dual awareness in meditation. Ann. N. Y. Acad. Sci. 1307, 9–18. doi: 10.1111/nyas.12261 24033505

[B25] Krishnamurti J. (1954). The first and Last Freedom. New York, NY: Harper & Row.

[B26] Krishnamurti J. (1969). Freedom from the known ed. M. Lutyens. New York, NY: Harper & Row.

[B27] Krishnamurti J. (1970). The Urgency of Change. New York, NY: Harper & Row.

[B28] Krishnamurti J. (1973). The Awakening of Intelligence. New York, NY: Harper & Row.

[B29] Krishnamurti J. (2000). Choiceless Awareness: A Study Book of the Teachings of J. Krishnamurti. Chennai: Krishnamurti Publications.

[B30] Lau M. A. Bishop S. R. Segal Z. V. Buis T. Anderson N. D. Carlson L. . (2006). The toronto mindfulness scale: development and validation. J. Clin. Psychol. 62, 1445–1467. doi: 10.1002/jclp.20326 17019673

[B31] Loy D. R. (1982). Enlightenment in Buddhism and Advaita Vedanta. Int. Philos. Q. 22, 65–74. doi: 10.5840/ipq19822217

[B32] Lutz A. Jha A. P. Dunne J. D. Saron C. D. (2015). Investigating the phenomenological matrix of mindfulness-related practices from a neurocognitive perspective. Am. Psychol. 70, 632–658. doi: 10.1037/a0039585 26436313 PMC4608430

[B33] Nolen-Hoeksema S. Morrow J. (1991). A prospective study of depression and distress following a natural disaster: the 1989 Loma Prieta earthquake. J. Pers. Soc. Psychol. 61, 115–121. doi: 10.1037/0022-3514.61.1.115 1890582

[B34] Nolen-Hoeksema S. Wisco B. E. Lyubomirsky S. (2008). Rethinking rumination. Perspect. Psychol. Sci. 3, 400–424. doi: 10.1111/j.1745-6924.2008.00088.x 26158958

[B35] Northoff G. (2025). The default mode network and inner time consciousness. Curr. Opin. Behav. Sci. 63:101524. doi: 10.1016/j.cobeha.2025.101524

[B36] Northoff G. Heinzel A. de Greck M. Bermpohl F. Dobrowolny H. Panksepp J. (2006). Self-referential processing in our brain—A meta-analysis of imaging studies on the self. NeuroImage 31, 440–457. doi: 10.1016/j.neuroimage.2005.12.002 16466680

[B37] Radhakrishnan S. (Trans.). (1953). The principal Upanishads. George Allen & Unwin. Available online at: https://archive.org/details/in.ernet.dli.2015.148291 (Accessed January 25, 2017).

[B38] Rajagopal S. (2024). The spiritual philosophy of Advaita: basic concepts and relevance to psychiatry. Indian J. Psychiatry 66, 202–207. doi: 10.4103/indianjpsychiatry.indianjpsychiatry_368_23 38523763 PMC10956581

[B39] Rao A. V. (1995). J. Krishnamurti's teachings: relevance to mental health. Indian J. Psychiatry 37, 155–160. 21743741 PMC2972428

[B40] Schacter D. L. Addis D. R. Hassabis D. Martin V. C. Spreng R. N. Szpunar K. K. (2012). The future of memory: remembering, imagining, and the brain. Neuron 76, 677–694. doi: 10.1016/j.neuron.2012.11.001 23177955 PMC3815616

[B41] Shiah Y.-J. (2016). From self to non-self: the non-self theory. Front. Psychol. 7:124. doi: 10.3389/fpsyg.2016.00124 26869984 PMC4740732

[B42] Siderits M. (2003). Personal Identity and Buddhist Philosophy: Empty persons. Burlington, VT: Ashgate.

[B43] Simeon D. Abugel J. (2006). Feeling unreal: Depersonalization disorder and the loss of the self . New York, NY: Oxford University Press. doi: 10.1093/oso/9780195170221.001.0001

[B44] Skowronski J. J. Sedikides C. (2019). On the evolution of the human self: a data-driven review and reconsideration. Self Identity 18, 4–21. doi: 10.1080/15298868.2017.1350601

[B45] Spreng R. N. Grady C. (2010). Patterns of brain activity supporting autobiographical memory, prospection, and theory of mind, and their relationship to the default mode network. J. Cogn. Neurosci. 22, 1112–1123. doi: 10.1162/jocn.2009.21282 19580387

[B46] Thibaut G. (Trans.). (1904). The Vedānta-Sutras with the commentary of Saṅkara. Oxford: Clarendon Press.

[B47] Thompson E. (2015). Waking, Dreaming, Being: Self and Consciousness in Neuroscience, Meditation, and Philosophy. New York, NY: Columbia University Press.

[B48] Vago D. R. Silbersweig D. A. (2012). Self-awareness, self-regulation, and self-transcendence (S-ART): a framework for understanding the neurobiological mechanisms of mindfulness. Front. Hum. Neurosci. 6:296. doi: 10.3389/fnhum.2012.00296 23112770 PMC3480633

[B49] Varela F. J. Thompson E. Rosch E. (2016). The Embodied Mind: Cognitive Science and Human Experience Rev. ed. Cambridge, MA: MIT Press.

[B50] Ward K. (2026). Modeling non-dual awareness via constraint closure: a reinterpretation of groundlessness. Neurosci. Conscious. 2026:niaf068. doi: 10.1093/nc/niaf068 41567792 PMC12817218

[B51] Wong P. T. P. Arslan G. Bowers V. L. Peacock E. J. Kjell O. N. E. Ivtzan I. . (2021). Self-transcendence as a buffer against COVID-19 suffering: the development and validation of the self-transcendence measure-B. Front. Psychol. 12:648549. doi: 10.3389/fpsyg.2021.648549 34690853 PMC8527188

[B52] Yaden D. B. Haidt J. Hood R. W. Vago D. R. Newberg A. B. (2017). The varieties of self-transcendent experience. Rev. Gen. Psychol. 21, 143–160. doi: 10.1037/gpr0000102

